# Adipose-derived stem cell extracellular vesicles attenuate liver fibrosis via restoration of gut barrier function and modulation of gut microbiota

**DOI:** 10.20517/evcna.2025.95

**Published:** 2025-11-24

**Authors:** Baitong Wu, Jingyi Guo, Jian Wang, Jiuxing Feng, Jun Xu

**Affiliations:** ^1^East Hospital, Stem Cell Research Center, School of Medicine, Tongji University, Shanghai 200120, China.; ^2^Shanghai Key Laboratory of Medical Epigenetics, Institutes of Biomedical Sciences, Fudan University, Shanghai 200032, China.; ^3^Liver Cancer Institute, Zhongshan Hospital, Fudan University, Shanghai 200032, China.; ^#^The authors contributed equally to this work.

**Keywords:** Liver fibrosis, ADSC-derived extracellular vesicles, gut microbiota, intestinal barrier, butyric acid, inflammation

## Abstract

**Aim:** Liver fibrosis (LF) is a major pathological stage that may progress to end-stage chronic liver injury but currently lacks effective treatment strategies. Previous studies have shown that adipose-derived stem cell extracellular vesicles (ADSC-EVs) play crucial roles in tissue repair, immune regulation, and anti-inflammatory effects. This study aims to elucidate the therapeutic effect of ADSC-EVs in LF and reveal their regulation mechanisms in gut-liver axis dysregulation.

**Methods:** The LF mouse model was established by intraperitoneal injection of diethylnitrosamine/CCl_4_. LF mice for ADSC-EV treatment received ADSC-EVs (200 μg per mouse) twice a week for three weeks. Then, hepatic function tests, liver and gut histopathology, and gut microbiota analyses were performed.

**Results:** ADSC-EVs effectively improved hepatic function, reduced collagen deposition and suppressed hepatic stellate cell activation, exhibiting potent anti-fibrotic potential in LF mice. Additionally, they significantly restored intestinal barrier integrity by reducing intestinal permeability and reinforcing the mucus barrier. Furthermore, ADSC-EV treatment regulated gut microbiota dysbiosis, increased the abundance of beneficial intestinal bacteria such as *Akkermansia muciniphila*. ADSC-EV intervention also elevated the level of butyric acid in cecal contents and significantly reduced systemic inflammation.

**Conclusion:** Our findings suggest that ADSC-EVs represent a promising novel therapeutic strategy for LF, promoting liver tissue repair, enhancing intestinal barrier function, and maintaining gut homeostasis to establish a virtuous circle within the liver-gut axis.

## INTRODUCTION

Liver fibrosis (LF) is an aberrant pathological wound-healing response to chronic liver injury, characterized by excessive deposition of extracellular matrix (ECM), which may subsequently develop into cirrhosis and hepatocellular carcinoma^[1,2]^. It is a major pathology status of chronic hepatitis, alcohol-related liver disease, and non-alcoholic fatty liver disease (NAFLD), with an increasing prevalence worldwide^[3]^. Its pathogenesis mainly involves the activation of hepatic stellate cells (HSCs), and the proliferation of ECM-producing myofibroblasts, driven by persistent inflammation and cytokines^[4]^. Currently, LF drugs primarily aim to eliminate pathogenic factors such as viruses, ethanol, and metabolic dysfunction, and restore tissue lesions^[5,6]^. Due to the complex etiology of LF, currently approved clinical drugs remain limited to resmetirom for NAFLD. Clinical candidate drugs such as irbesartan and cenicriviroc have reported failure in clinical trials^[7]^. Thus, effective therapeutic strategies for LF remain a critical unmet need.

Numerous studies indicated that the LF process generally had varying degrees of intestinal barrier dysfunction, such as abnormal tight junction protein expression, leading to altered intestinal permeability; and reduced goblet cell number or function, resulting in a thinning of the intestinal mucus layer, finally with a weakening of the physical barrier function. Alterations of the intestinal barrier might contribute to exacerbated immune responses in liver diseases^[8]^. Increased intestinal permeability permits metabolites or pathogen-associated molecular patterns (PAMPs) to translocate to the liver via the portal circulation, where interaction with pattern recognition receptors (PRRs) on hepatic-resident cells, potentially inducing or exacerbating detrimental immune responses^[9]^. Additionally, the progression of chronic liver disease is associated with gut microbiota imbalance. It has been reported that the gut microbiome in metabolic dysfunction-associated steatotic liver disease (MASLD) was featured with an enrichment of *Proteobacteria*, *Escherichia*, *Clostridium* and *Streptococcus*, along with a reduced abundance of beneficial commensals such as *Firmicutes*, *Eubacterium* and *Faecalibacterium*, implying a reduced capability of intestinal protection and anti-inflammatory function^[10]^. There was also gut microbial dysbiosis in LF, including dysregulated composition and function of the gut microbiome. Consequently, integrating intestinal modulatory strategies into anti-fibrotic protocols was critical for achieving maximal treatment benefits.

Extracellular vesicles (EVs), as nanoscale extracellular lipid bilayer vesicles, contain diverse bioactive molecules, such as proteins, nucleic acids, lipid secreted complexes, *etc.*, and they play a crucial role in intercellular communication^[11,12]^. Several studies showed that mesenchymal stem cell-derived EVs (MSC-EVs) exhibited therapeutic potential in repairing tissue injuries, including renal failure^[13]^, acute tubular injury^[14,15]^ and skin damage^[16]^. Adipose-derived stem cells (ADSCs) offered the advantages of abundant sources, easy accessibility and higher proliferation *in vitro*, making them an ideal seed cell in regenerative medicine. Studies showed that MSC-EV therapy could reduce the hepatic fibrotic encapsulation, attenuate hepatic inflammation, and decrease collagen deposition in CCl_4_-induced fibrosis^[17]^. Our previous studies also demonstrated the anti-inflammatory, antioxidant, and tissue-repair functions of ADSC-EVs in an acute liver failure model^[18]^.

In this research, we aimed to elucidate the therapeutic effect of ADSC-EVs in LF and revealed their regulation mechanisms in gut-liver axis dysregulation. Diethylnitrosamine (DEN)/CCl_4_ was used to establish the LF mouse model. Hepatic function, liver and gut histopathology, and gut microbiota of mice in each group were evaluated to assess the effects of ADSC-EVs. Our findings demonstrated that ADSC-EVs not only exhibited potent anti-fibrotic potential but also restored intestinal barrier integrity in LF mice. Additionally, ADSC-EV treatment improved gut microbiota dysbiosis by increasing beneficial bacteria such as *Akkermansia muciniphila* (*A. muciniphila*) and elevating butyric acid levels, significantly reducing systemic inflammation, making it a promising novel therapeutic strategy for LF.

## METHODS

### Reagents

CCl_4_ was purchased from MedChemExpress (MCE) Chemical Reagent (HY-Y0298, USA). N-Nitrosodiethylamine (DEN) was purchased from Meilunbio® Co., Ltd. (MB4816-1, Dalian, China).

### Preparation of ADSCs and ADSC-EVs

Isolation and culture of primary ADSCs and isolation of ADSC-EVs were performed according to the established method in our previous study^[19]^. ADSCs were cultured in Dulbecco’s Modified Eagle Medium/Nutrient Mixture F-12 (DMEM-F12, Gibco, USA) supplemented with 10% fetal bovine serum (FBS, Gibco, USA), 10 ng/mL basic fibroblast growth factor (bFGF, Novoprotein, China), and 1% penicillin-streptomycin solution (10,000 U/mL, Gibco, USA) at 37 °C, 5% CO_2_. The basic culture medium was replaced with EV-free FBS complete medium 24 h before supernatant collection. The supernatant was then collected, and ADSC-EVs were isolated by differential centrifugation according to our established protocol.

### Characteristic of isolated ADSC-EVs

EV concentration, particle size and distribution were quantified using nanoparticle tracking analysis (NTA, NanoSight300, Malvern Instruments Ltd, UK). Briefly, samples were diluted with 1 mL phosphate-buffered saline (PBS) to an optimal concentration, and then loaded into the cubicle. Particles were then tracked and illuminated by laser light under Brownian motion. Samples were measured with particle concentration, size distribution, scatter intensity and calculated based on the Stokes-Einstein equation.

The ultrastructure of EVs was characterized by transmission electron microscopy (TEM) using a Hitachi HT7800 electron microscope (Hitachi, Japan).

Fluorescent-labeled antibodies (FITC, PC5) targeting EVssomal surface markers CD63 (NHA063-A647, Fuliu, China), CD81 (NHA081-FITC, Fuliu, China), and CD9 (NHA009-A488, Fuliu, China) were incubated with EVs at 4 °C for 60 min in the dark. Control groups were established to exclude non-specific antibodies and auto-fluorescence. Processed samples were subjected to instrument detection at a flow rate of 35 nL/min (NanoFCM SNA-D1, Fuliu, China).

### Animals

Specific pathogen-free C57/BL6 mice (Male, 3 weeks of age) were purchased from Shanghai Slack Laboratory Animal Co., Ltd. (Shanghai, China). All animal experiments and research protocols were approved by the Ethical Committee of Laboratory Animals Research Center of Tongji University (Approval No.: TJAA07624403).

### Experimental design

To investigate ADSC-EV therapeutic effect in the LF mouse model, mice were randomly divided into three groups (*n* = 8 mice per group), including the Sham group (healthy controls without LF induction), the LF group (LF) and the liver fibrosis + ADSC-EV group (LF + EV). For the LF model, 3-week-old male mice were injected intraperitoneally (*i.p.*) with DEN (20 mg/kg) once a week for two weeks, followed by intraperitoneal injections of CCl_4_ (2 mL/kg) three times per week during weeks 3-6 and twice per week during weeks 7-9. Mice in the Sham group received dimethyl sulfoxide (DMSO) or oil injections instead of DEN or CCl_4_. Mice in the LF + EV group received ADSC-EVs (200 μg per mouse, intravenously via tail vein) twice a week for three weeks from weeks 7 to 9.

### Histological analysis

Fresh mouse liver or intestinal tissues were fixed in 4% paraformaldehyde (≥ 24 h), dehydrated through an ethanol gradient, embedded in paraffin, and sectioned at a thickness of 4 μm. Consecutive sections were processed for hematoxylin and eosin (H&E) staining (C0105S, Beyotime, China), Sirius Red staining (60415ES50, Yeasen Biotechnology, China), and Periodic Acid-Schiff (PAS) staining (C0142S, Beyotime, China) following standard protocols. Immunohistochemistry (IHC) staining for alpha smooth muscle actin (α-SMA), zonula occludens-1 (ZO-1), and myeloperoxidase (MPO) was also performed according to standard protocols. Primary antibodies included: Anti-α-SMA (ab124964, Abcam, USA), Anti-ZO-1 tight junction protein (ab221546, Abcam, USA), Anti-MPO (AG2657, Beyotime, China). Secondary antibodies included horseradish peroxidase (HRP)-conjugated goat anti-rabbit immunoglobulin G (IgG) (H + L) (A0208, Beyotime, China).

### Quantitative real-time polymerase chain reaction assay

Total RNA was extracted from freshly dissected mouse intestinal tissues using TRIzol® Reagent (Thermo Fisher Scientific, USA) according to the manufacturer’s protocol. Complementary DNA (cDNA) was synthesized from 1 µg of total RNA using the 1st Strand cDNA Synthesis Kit (Vazyme, China). Quantitative real-time polymerase chain reaction (PCR) (qRT-PCR) was performed using ChamQ Universal SYBR® quantitative PCR (qPCR) Master Mix (Vazyme, China) on a Light Cycler96 real-time PCR system. Glyceraldehyde-3-phosphate dehydrogenase (GAPDH) messenger RNA (mRNA) was used as an internal control. The primer sequences used in this experiment were as follows: mouse ZO-1 Forward, 5′-GCCGCTAAGAGCACAGCAA-3′; mouse ZO-1 Reverse, 5′-TCCCCACTCTGAAAATGAGGA-3′; mouse Occludin Forward, 5′-TTGAAAGTCCACCTCCTTACAGA-3′; mouse Occludin Reverse, 5′-CCGGATAAAAAGAGTACGCTGG-3′; mouse Claudin-3 Forward, 5′-ACCAACTGCGTACAAGACGAG-3′; mouse Claudin-3 Reverse, 5′-CAGAGCCGCCAACAGGAAA-3′; mouse Mucin (Muc)-1 Forward, 5′-GGCATTCGGGCTCCTTTCTT-3′; mouse Muc-1 Reverse, 5′-TGGAGTGGTAGTCGATGCTAAG-3′; mouse Muc-2 Forward, 5′-AGGGCTCGGAACTCCAGAAA-3′, mouse Muc-2 Reverse, 5′-CCAGGGAATCGGTAGACATCG-3′; mouse GAPDH Forward, 5′-CGGAGTCAACGGATTTGGTCGTAT-3′; and mouse GAPDH Reverse, 5′-GCCTTCTCCATGGTGGTGAAGAC-3.

### 16S rRNA sequencing

Intestinal contents were collected from the Sham group, LF-1W group, LF-1W-EV group, the LF-3W group, and the LF-3W-EV group for DNA extraction. The V3-V4 variable region of the 16S ribosomal RNA (rRNA) gene was amplified by PCR using specific primers (341F/806R). Then, the amplified product was purified using magnetic beads, and a sequencing library was constructed using Illumina TruSeq Nano DNA LT Library Prep Kit for further sequencing (Illumina MiSeq platform). The result was analyzed at the Omicsmart platform (https://www.omicsmart.com).

### Detection of butyric acid level in intestinal contents

The butyric acid level in cecal contents was measured using a commercial enzyme-linked immunosorbent assay (ELISA) kit (HB142-SH, Hengyuan, China) via a competition method following the manufacturer’s protocol.

### Western blot analysis

Protein extraction from both cultured cells and liver tissue homogenates was performed using radio-immunoprecipitation assay (RIPA) lysis buffer supplemented with 1% protease inhibitor (Epizyme, China). Protein concentration was quantified using a bicinchoninic acid (BCA) assay (Epizyme, China). Protein samples mixed with loading buffer (Beyotime, China) were denatured by boiling, separated by sodium dodecyl sulfate polyacrylamide gel electrophoresis (SDS-PAGE), and transferred onto polyvinylidene fluoride (PVDF) membranes (Millipore, USA) via Western blotting. After blocking non-specific binding with 5% skim milk (Solarbio, China), membranes were incubated overnight at 4 °C with primary antibodies. Following washing with Tris-Buffered Saline and Tween 20 (TBST, Solarbio, China), membranes were incubated with secondary antibody for 1 h at room temperature, washed again, and bands were detected using electrochemiluminescence (ECL) reagent (Abcam, USA) and imaged. Primary antibodies included Anti-CD9 (A19027, Abclonal, China), Anti-CD81 (56039, CST, USA), Anti-ALIX (92880S, CST, USA), Anti-tumor susceptibility gene 101 (TSG101) (72312, CST, USA), Anti-Calnexin (A4846, Abclonal, China), Anti-α-SMA (ab124964, Abcam, USA), and Anti-GAPDH (60004-1-Ig, Proteintech, China). Secondary antibodies included HRP-conjugated goat anti-mouse IgG (H + L) (A0216, Beyotime, China) and HRP-conjugated goat anti-rabbit IgG (H + L) (A0208, Beyotime, China).

### Serum biochemical analysis

Before sacrificing the mice, blood was collected and centrifuged at 4,000 × *g* for 15 min at room temperature to obtain serum for subsequent biochemical analysis. Serum levels of alanine aminotransferase (ALT) and aspartate aminotransferase (AST) for hepatic function were measured using an automatic biochemical detection instrument (Rayto, China). Serum levels of D-Lactate and diamine oxidase (DAO), markers of intestinal barrier function, were determined by ELISA according to the manufacturer’s instructions. Commercial reagent kits for mouse serum D-Lactate (SBJ-M0727, Senbeijia, China) and DAO (SBJ-M0211, Senbeijia, China) were used.

### Hepatic monocyte-derived macrophage analysis by flow cytometry

The detailed procedure followed the comprehensive analysis of liver macrophage composition by flow cytometry in murine non-alcoholic steatohepatitis (NASH)^[20]^. Briefly, the mice were perfused with PBS through the portal vein, and the liver was then transferred into a digestion buffer [consisting of DMEM medium supplemented with 0.75 mg/mL Collagenase A and 50 μg/mL deoxyribonuclease I (DNase I), Sigma, USA]. The tissue was incubated at 37 °C for 30 min to allow enzymatic digestion. After digestion, enzyme-containing DMEM was removed by centrifugation at 50 × *g* for 1 min, followed by 900 rpm for 7 min at 4 °C to obtain a single-cell suspension for flow cytometry. The cells were resuspended and stained in 100 μL of antibody cocktail on ice and in the dark for 45 min. The antibodies included peridinin chlorophyll protein (PerCP)/Cyanine7 anti-CD45 (103132, BioLegend, USA); BV421 anti-lymphocyte antigen 6 family member G (Ly6G, 127628, BioLegend, USA); KIRAVIA Blue 520 CD11c (117364, BioLegend, USA); Allophycocyanin (APC) anti-CD3 (100236, BioLegend, USA); APC/Cyanine7 anti-CD11b (101226, BioLegend, USA); PE anti-F4/80 (123110, BioLegend, USA); phycocerythrin (PE)/Cyanine7 anti-Ly6c (12807, BioLegend, USA). Then, the cells were washed with fluorescence-activated cell sorting (FACS) buffer, pelleted at 650 × *g* for 4 min at 4 °C, and filtered with a 70-μm cell strainer and prepared for analysis on a flow cytometer.

### Statistical analysis

All data are shown as the mean ± standard deviation (SD). Comparisons between two groups were performed using the unpaired Student’s *t*-test. *P*-value < 0.05 was considered statistically significant. Analyses were performed using GraphPad Prism 8.3.0 software. Flow cytometry data were analyzed using FlowJo 10.8.1.

## RESULTS

### Preparation and identification of ADSC-EVs

**Figure 1:**
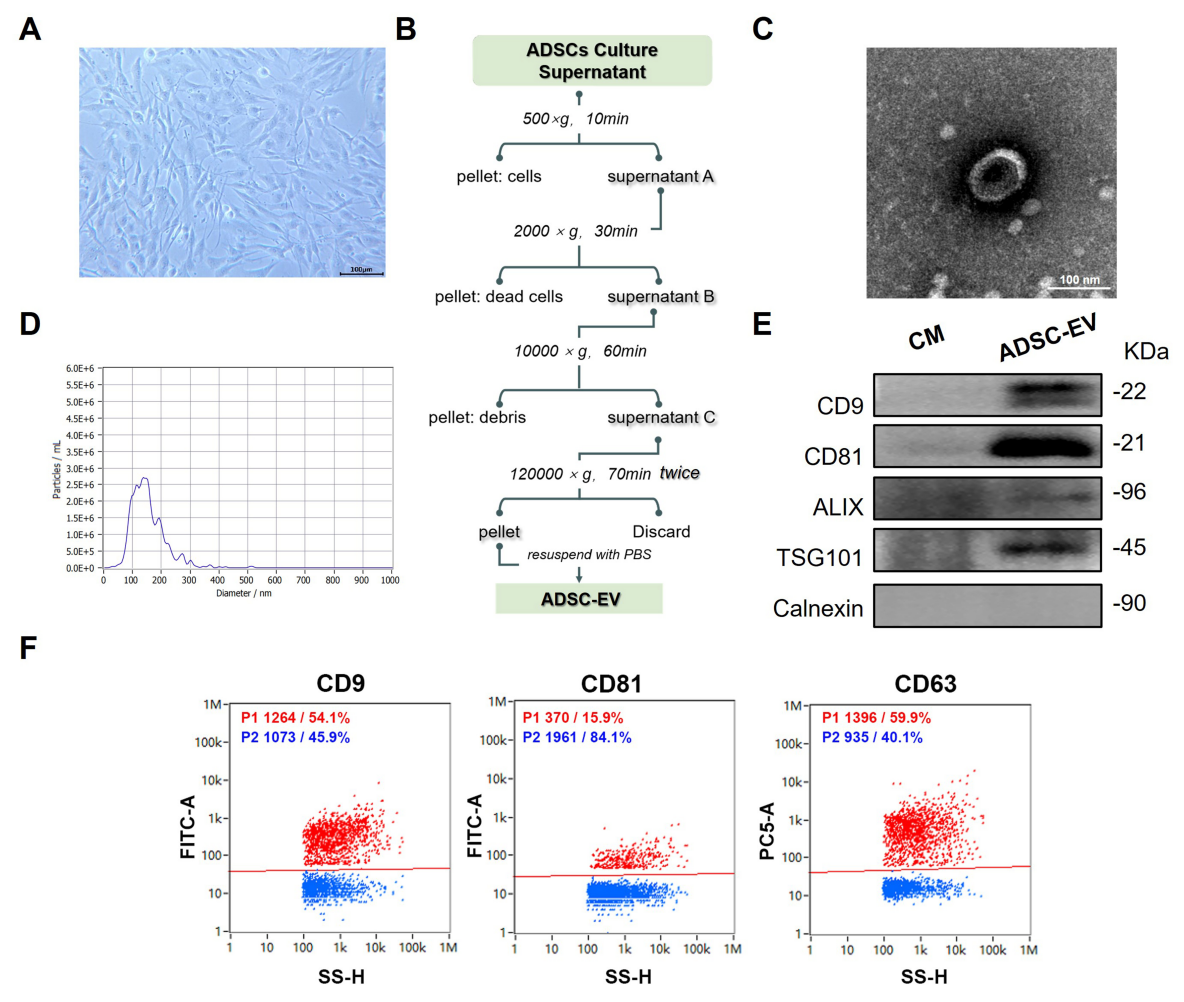
Preparation and characterization of ADSC-EV. (A) Morphology of primary ADSCs at passage 4 (P4) at 80% confluence (scale bar: 100 µm); (B) Schematic workflow for isolating ADSC-EV from the ADSC culture supernatant; (C) Representative TEM image of isolated ADSC-EV (scale bar: 100 nm); (D) NTA of ADSC-EV; (E) Immunoblots for typical EV markers (CD9, CD81, TSG101, Alix) and negative marker (Calnexin) in isolated ADSC-EV and conditional CM; (F) NanoFCM of isolated ADSC-EV for positive markers CD9, CD81, and CD63. ADSC-EV: Adipose-derived stem cell extracellular vesicle; TEM: transmission electron microscopy; NTA: nanoparticle tracking analysis; CM: culture medium; NanoFCM: nano-flow cytometry analysis.

Human ADSCs were isolated and cultured as described in a previous study ^[21]^. As shown in Figure 1A, ADSCs exhibited a similar typical morphology to mesenchymal stem cells (MSCs), characterized by adherent, fibroblast-like, and spindle-shaped appearance with elongated processes and a large central nucleus. Then, ADSC-EVs were isolated from conditioned media of Passage 3-Passage 5 ADSCs using differential ultracentrifugation, following established protocols with modifications [Figure 1B]. TEM analysis showed that ADSC-EVs were spherical with a lipid bilayer membrane and a typical cup-shaped or saucer-like concavity [Figure 1C]. The size distribution and concentration of EVs were quantified using NTA [Figure 1D]. The majority population of vesicles (93.4%) fell within the accepted size range (30-200 nm) and the concentration was 6.2 × 10^9^ particles/mL. Western blot analysis confirmed that the ADSC-EV exhibited enrichment of canonical EV markers, including CD9, CD81, Alix, and Tsg101, whereas it lacked the endoplasmic reticulum marker Calnexin [Figure 1E]. Additionally, nano-flow cytometry analysis (NanoFCM) revealed that ADSC-EVs expressed CD9 (54.1%), CD81 (15.9%), and CD63 (59.9%) [Figure 1F].

### ADSC-EVs alleviate DEN/CCl_4_-induced hepatic fibrosis in mice

**Figure 2:**
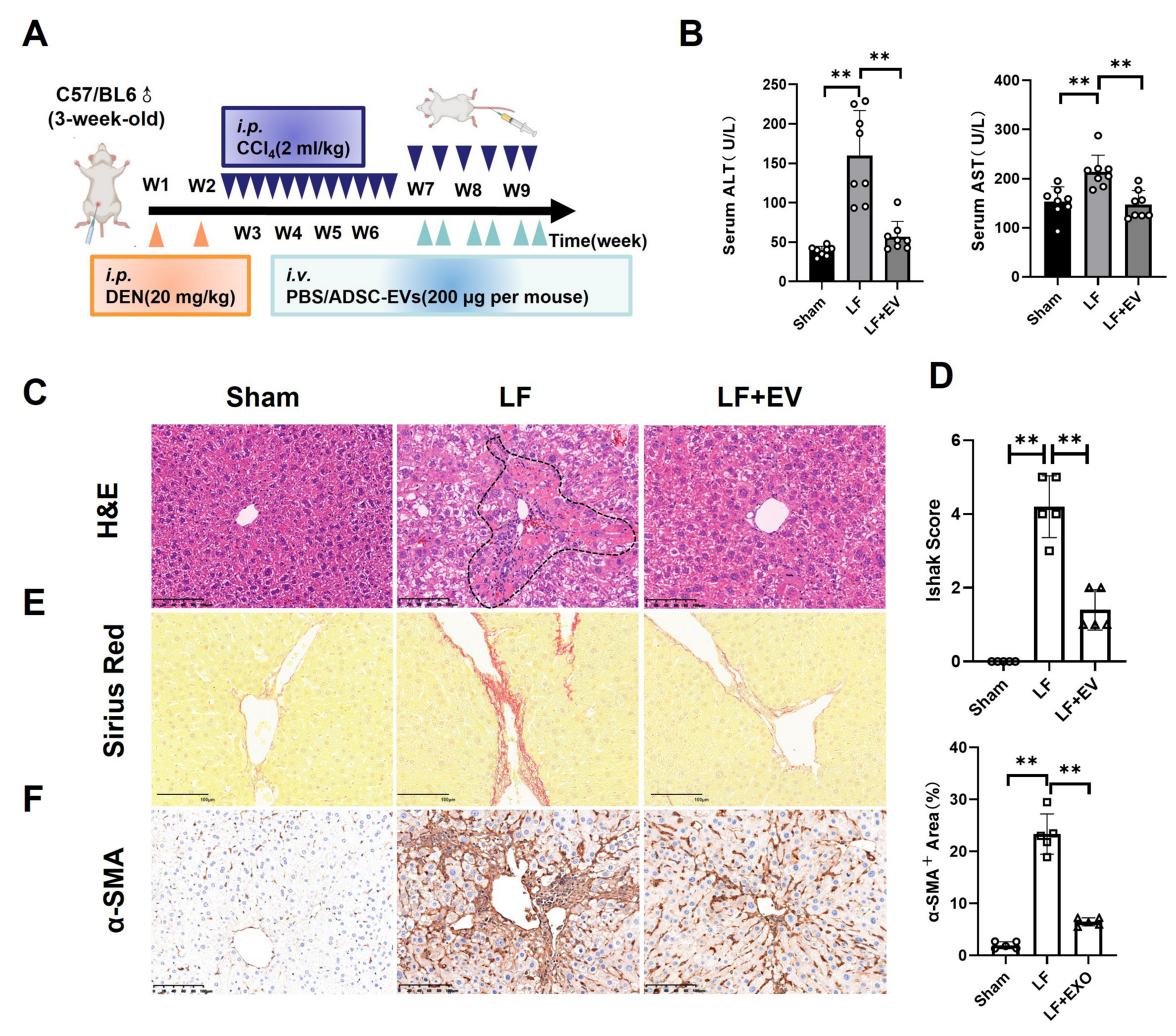
ADSC-EVs inhibit DEN/CCl_4_-induced hepatic fibrosis in mice. (A) Schematic diagram of DEN/CCl_4_-induced LF animal model establishment (*i.p.*) and ADSC-EV treatment strategy (*i.v.*); (B) The biochemical parameters measurement of ALT and AST in the mouse serum of each group (*n* = 8); (C) Representative histomorphology images of liver tissues by H&E staining (scale bar: 100 µm). The dotted line indicates the severely damaged area; (D) LF stage assessed using the Ishak score system (*n* = 5); (E) Representative images of liver tissues by Sirius Red staining (scale bar: 100 µm); (F) Expression of the activated HSCs marker α-SMA in liver tissues by IHC staining (scale bar: 100 µm). All data are shown as the mean ± SD. ^**^P < 0.01. ADSC-EVs: Adipose-derived stem cell extracellular vesicles; DEN: diethylnitrosamine; LF: liver fibrosis; *i.p.*: intraperitoneal injection; *i.v.*: intravenous injection; ALT: alanine aminotransferase; AST: aspartate aminotransferase; H&E: hematoxylin and eosin; HSCs: hepatic stellate cells; α-SMA: alpha smooth muscle actin; IHC, immunohistochemistry; SD: standard deviation; PBS: phosphate-buffered saline; LF + EV: liver fibrosis + ADSC-EV group.

To determine the therapeutic effect of ADSC-EVs in LF, we employed an LF model in which C57BL/6 mice received DEN (*i.p.*) once a week for two weeks, followed by CCl_4_ (2 mL/kg) three times a week for weeks 3-6 and 2 times a week for weeks 7-9, resulting in severe LF [Figure 2A]. Meanwhile, the LF + EV group was treated with ADSC-EVs (200 μg/mice) twice a week for three weeks. After the 3-week ADSC-EV treatment period, serum was collected from each group, and the mice were sacrificed to harvest liver tissue for further functional assessment.

Serum biochemical analysis revealed that exposure to DEN/CCl_4_ elevated ALT and AST levels in LF group mice, while ADSC-EV treatment significantly attenuated these elevations, indicating marked recovery of liver function [Figure 2B]. Then, the histopathological analysis of H&E staining demonstrated that, compared with the liver section in the LF group, LF + EV mice showed markedly reduced cellular edema, the areas of centrilobular necrosis, inflammatory cell infiltration, and ballooning degeneration [Figure 2C]. This improvement was a quantitative analysis using Ishak fibrosis scores, with significantly lower scores in ADSC-EV-treated mice [Figure 2D]. As is known, LF pathology was characterized by excessive deposition of fibrillar collagen and other ECM components, driven by activated HSCs that transformed into proliferative, contractile, fibrogenic myofibroblasts. Therefore, we evaluated the collagen deposition level by Sirius Red staining and HSC activation by IHC of α-SMA. Sirius Red staining showed a robust reduction in collagen deposition and bridging fibrosis, confirming the anti-fibrotic efficacy of ADSC-EVs [Figure 2E]. Critically, IHC staining revealed a significant decrease of α-SMA^+^ area portion compared to the LF control, indicating HSC activation [Figure 2F]. These findings suggested that ADSC-EVs could alleviate DEN/CCl_4_-induced hepatic fibrosis.

### ADSC-EVs restore the intestinal barrier integrity through reduced permeability and reinforced mucus barrier in mice with LF

**Figure 3:**
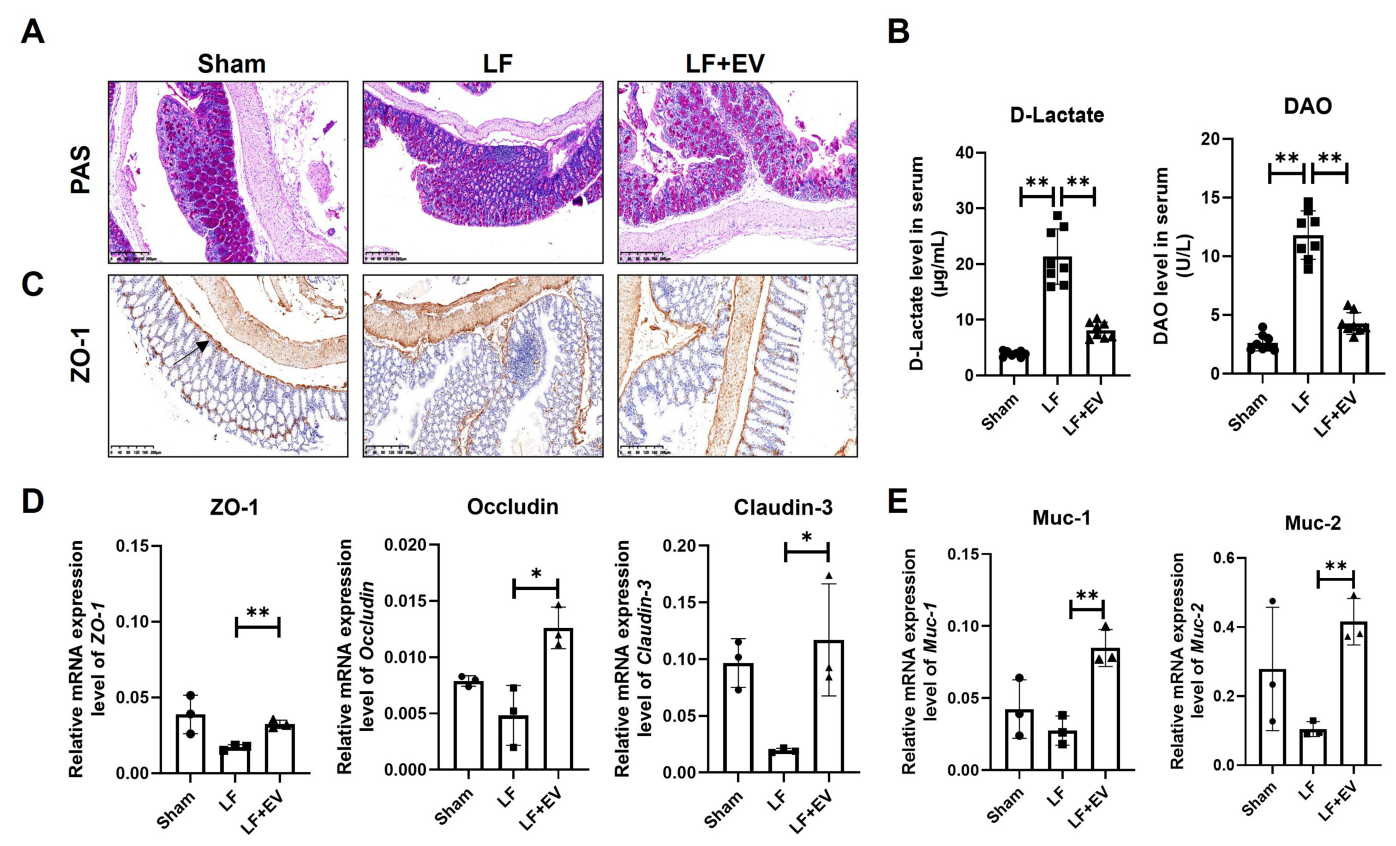
ADSC-EVs improve the intestinal barrier integrity by reducing intestinal permeability and reinforcing the mucus barrier in mice with LF. (A) PAS staining of colonic mucosa in Sham, LF and LF + EV group mice (scale bar: 200 µm); (B) The intestinal injury marker D-Lactate and DAO level in the serum of mice in each group (*n* = 8); (C) Representative images of ZO-1 expression in liver tissues by IHC staining (scale bar: 200 µm); (D) mRNA levels of tight junction associated genes ZO-1, Occludin, and Claudin-3 in colonic tissue were evaluated by q-PCR (*n* = 3); (E) The mRNA levels of mucosal barrier-associated genes Muc-1 and Muc-2 in colonic tissue were evaluated by q-PCR (*n* = 3). The expression was normalized to Gapdh. All data are shown as the mean ± SD. ^*^P < 0.05; ^**^P < 0.01. ADSC-EVs: Adipose-derived stem cell extracellular vesicles; LF: liver fibrosis; PAS: periodic acid Schiff; DAO: diamine oxidase; ZO-1: zonula occludens-1; IHC: immunohistochemistry; Occludin: tight junction protein Occludin; Claudin-3: tight junction protein 3; q-PCR: quantitative polymerase chain reaction; Muc-1: Mucin 1; Muc-2: Mucin 2; SD: standard deviation; LF + EV: liver fibrosis + ADSC-EV group.

As shown in Figure 3A, PAS staining revealed that colon tissue in the Sham group was intact, with normal crypt architecture, preserved goblet cells, and only scattered inflammatory cells in the lamina propria. However, the colon tissue in the LF group exhibited significant damage to crypt structures, a reduction in the number of goblet cells, and a large number of inflammatory cells infiltrated between the mucosa and submucosa. By administering ADSC-EVs, the morphology of colon tissue was improved in terms of mucosal damage, crypt structure, the number of intestinal epithelium and goblet cells and inflammation. Then, we measured two critical biomarkers, D-lactate and DAO, to evaluate gut permeability. Generally, when the tight junctions of the intestinal epithelium are disrupted by factors such as portal hypertension or inflammation, D-lactate passively diffuses from the intestinal lumen into the bloodstream, resulting in increased serum levels^[22]^. DAO, mainly exists in the cytoplasm of mature intestinal epithelial cells, is responsible for catalyzing the oxidative deamination of biogenic amines such as histamine and putrescine^[23]^. When intestinal epithelial cells are in a state of necrosis or apoptosis, DAO is directly released into the blood. The serum biochemistry displayed that the abnormal increase of D-lactate and DAO in the LF group was suppressed by ADSC-EV treatment, indicating dual protection of both intestinal permeability and the integrity of the mucosal layer [Figure 3B].

To further evaluate the impact of ADSC-EVs on intestinal barrier integrity, we assessed the expression and localization of ZO-1, a critical tight junction protein, in intestinal tissues via IHC and q-PCR. IHC analysis revealed that intestinal tissues from the LF group exhibited disruption of the characteristic continuous linear ZO-1 expression along the apical membrane of intestinal epithelial cells. Instead, ZO-1 displayed a fragmented and discontinuous distribution, indicating the tight junction collapse and increased intestinal permeability. Administration of ADSC-EVs attenuated these pathological changes, effectively restoring the continuous linear expression of ZO-1 protein at the apical membrane of epithelial cells within both villi and crypts [Figure 3C]. Concomitantly, ADSC-EVs restored the mRNA expression of key tight junction-related genes, including ZO-1, Occludin, and *Claudin-3* [Figure 3D]. Also, ADSC-EVs could upregulate Muc-1 and Muc-2 gene mRNA expression, which, both relative to goblet cells, constitute the mucus layer that physically segregates microbiota from the intestinal epithelium, forming a mucus barrier [Figure 3E]. Collectively, these EVs showed a positive effect in repairing intestinal barrier dysfunction by reinforcing tight junction protein expression and reconstructing the mucus barrier.

### ADSC-EVs alter the gut microbiota disorder and increase the abundance of probiotic *A. muciniphila* in mice with LF

**Figure 4:**
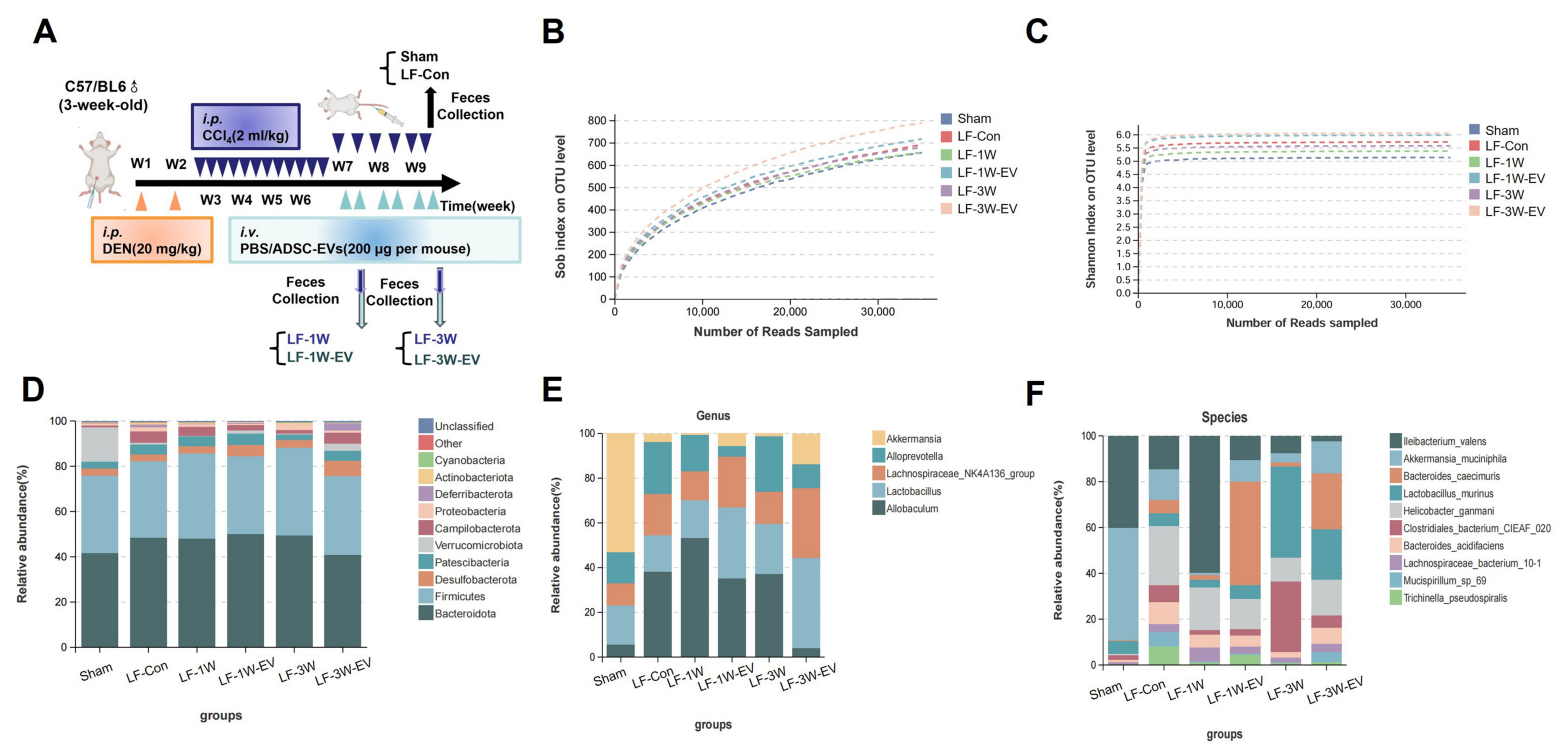
ADSC-EVs restore gut microbiota and increase the abundance of probiotic *A. muciniphila* in live fibrosis mice. (A) Schematic diagram of fecal samples collection for 16S rRNA sequencing, including the Sham group (*n* = 3), LF-Con (*n* = 5), LF-1W (*n* = 3), LF-1W-EV (*n* = 3), LF-3W (*n* = 3), LF-3W-EV (*n* = 3); Alpha diversity analysis based on (B) Sob index and (C) Shannon index, rarefaction curves indicate adequate sequencing depth; Relative abundance of gut microbiota at the (D) phylum, (E) genus and (F) species levels in each group of mice. All data are shown as the mean ± SD. ^*^P < 0.05; ^**^P < 0.01. ADSC-EVs: Adipose-derived stem cell extracellular vesicles; *A. muciniphila*: *Akkermansia muciniphila*; 16S rRNA: 16S ribosomal RNA; LF: liver fibrosis; SD: standard deviation; *i.p.*: intraperitoneal injection; DEN: diethylnitrosamine; *i.v.*: intravenous injection; PBS: phosphate-buffered saline; OTU: operational taxonomic units.

In addition to repairing the intestinal barrier, we speculated that ADSC-EV may also modulate the gut microbiota in LF mice. We therefore collected fecal samples from Sham, LF-Con, LF-1W, LF-1W-EV, LF-3W, and LF-3W-EV groups for 16s rRNA sequencing. The time points for sample collection in each group are illustrated in Figure 4A. Both the richness rarefaction curves (Sobs) and the Shannon rarefaction curves tend to flatten, indicating sufficient sequencing depth and sample capacity, which could cover most of the microbiota diversity [Figure 4B and C]. Then, we analyzed the sequencing data to evaluate microbiota composition. At the phylum level, the intestinal microbiota of mice in each group mainly include *Bacteroidota*, *Firmicutes*, *Desulfobacterota*, *Patescibacteria*, *Verrucomicrobiota, etc.* [Figure 4D], with *Bacteroidetes* and *Firmicutes* being the dominant bacterial phyla in the gut microbiota. We further characterized the effect of ADSC-EVs on the relative abundance of gut microbiota at the genus level. The general landscape of gut microbiota and differences in composition between ADSC-EV-treated and their control mice were shown at genus and species levels. At the genus level, the top five most abundant gut microbiota in mice included *Akkermansia*, *Allobaculum*, *Lactobacillus*, *Lachnospiraceae_NK4A136_group* and *Alloprevotella* [Figure 4E]. Compared with the Sham group, the relative abundance of *Akkermansia* was significantly decreased in the LF-Con, LF-1W and LF-3W groups; however, this reduction was reversed following one week and three weeks of ADSC-EV treatment. Also, ADSC-EV administration significantly increased the relative abundance of *Lachnospiraceae-NK4A136-group* in the gut microbiota compared with the LF-1W and LF-3W groups. By coincidence, both *Lachnospiraceae NK4A136-group* and *Akkermansia* are pivotal producers of short-chain fatty acid (SCFA), which maintain an acidic intestinal environment, inhibit the growth of pathogenic bacteria, and provide energy for intestinal epithelial cells^[24]^. To be specific, at the species level, the abundance of *A. muciniphila* was significantly decreased under LF stimulation, whereas it was significantly increased in ADSC-EV-treated mice [Figure 4F]. Some recent studies have shown that the decreased abundance of *A. muciniphila* is associated with the progression of LF and cirrhosis^[25,26]^. This bacterium exerts hepatoprotective effects by repairing the intestinal mucosal barrier, regulating immune inflammation, and improving metabolic homeostasis. Collectively, these results implied that ADSC-EVs restored the dysbiosis of the microbiota in LF mice by increasing the abundance of beneficial bacteria (e.g., *Akkermansia*, *Lachnospiraceae NK4A136-group*) and specifically elevating the levels of probiotic *A. muciniphila*, which may contribute to intestinal barrier repair and anti-fibrosis.

### Administration of ADSC-EVs elevates the butyric acid level of intestinal contents and decreases the systemic inflammation in mice with LF

**Figure 5:**
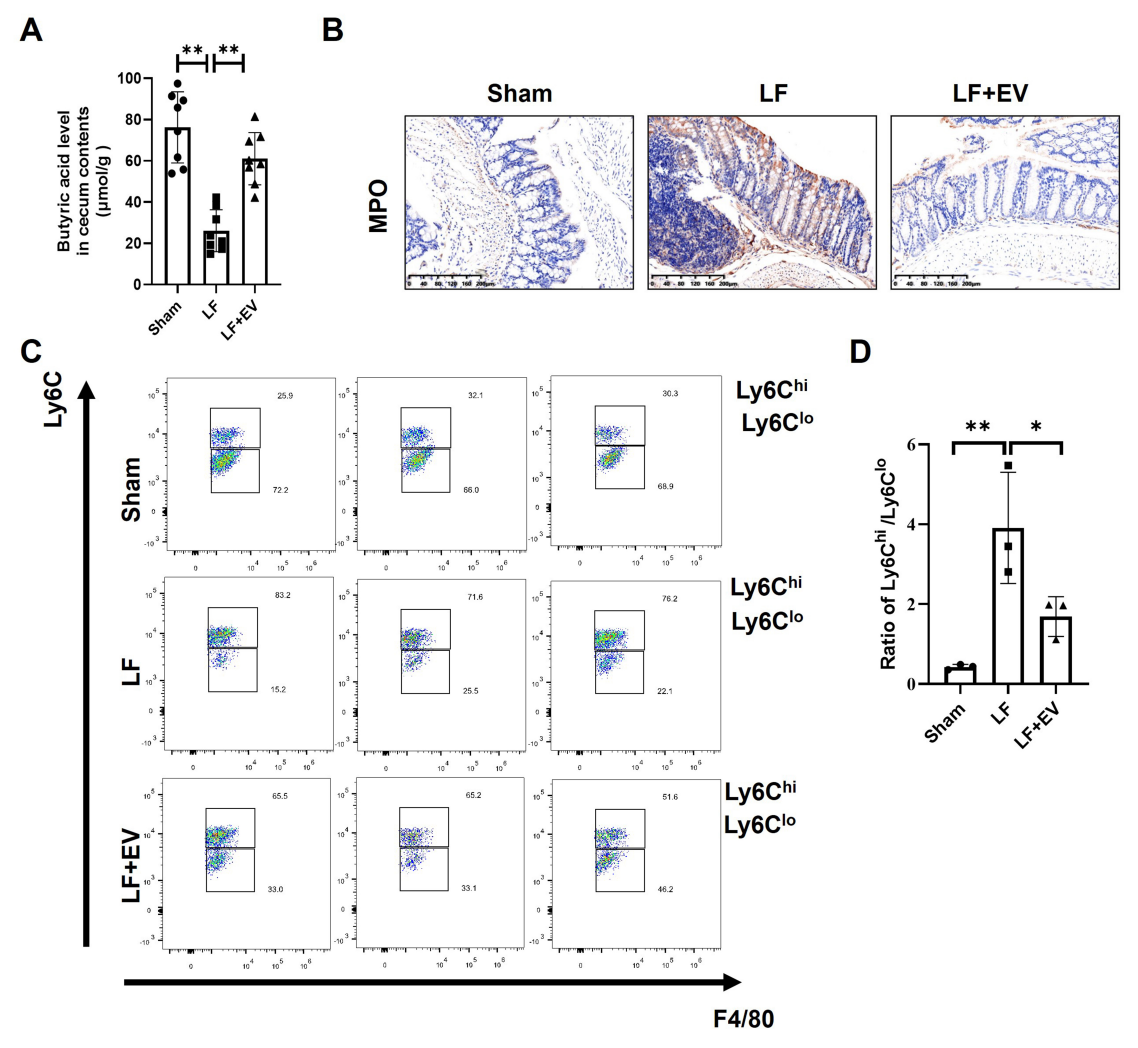
Administration of ADSC-EVs elevates the butyric acid level of intestinal contents and decreases the systemic inflammation in mice with LF. (A) Comparison of the butyric acid level in the cecal content from each group of mice (*n* = 8); (B) Representative images of MPO staining in the mouse colon tissues (scale bar: 200 µm); (C) The hepatic infiltrated MoMFs in mice with LF were analyzed by flow cytometry. The gating strategy for infiltrated inflammatory MoMFs was CD45^+^-Ly6G^-^-CD11c^-^-CD3^-^-CD11b^+^-F4/80^low^-Ly6c^high^ and for infiltrated restorative MoMFs was CD45^+^-Ly6G^-^-CD11c^-^-CD3^-^-CD11b^+^-F4/80^low^-Ly6c^low^. Also, (D) the result was quantified by the ratio of Ly6C^high^/Ly6C^low^ (*n* = 3). All data are shown as the mean ± SD. ^*^P < 0.05; ^**^P < 0.01. ADSC-EVs: Adipose-derived stem cell extracellular vesicles; LF: liver fibrosis; MPO: myeloperoxidase; MoMFs: monocyte-derived macrophages; SD: standard deviation; LF + EV: liver fibrosis + ADSC-EV group.

We previously demonstrated a significant increase in the abundance of *A. muciniphila* in the gut contents of LF mice. Generally, *A. muciniphila* can degrade intestinal mucins by cleaving protective glycans, thereby releasing oligosaccharides that are further metabolized into acetic acid and propionic acid. These metabolites subsequently stimulate the proliferation of butyrate-producing bacteria^[27]^. Butyric acid, a key microbial metabolite, not only serves as the primary energy source for colonic epithelial cells but also reinforces intestinal barrier integrity by upregulating mucin synthesis and tight junction proteins. More importantly, butyrate also plays a positive role in inflammation suppression, which enables it to both maintain intestinal homeostasis and inhibit systemic inflammation^[28]^. Thus, we further measured the butyric acid level in intestinal content by the ELISA assay. We found that the butyric acid level in the LF group was significantly decreased compared with the Sham group while ADSC-EV treatment led to a higher production of butyric acid in LF mice [Figure 5A]. Given the change of butyrate levels, we hypothesized that concomitant changes in systemic inflammation may occur in LF mice. MPO staining of intestinal tissue revealed a marked increase in neutrophil accumulation in LF mice, with neutrophils predominantly clustered around crypt abscesses, indicating severe inflammatory infiltration of the intestinal mucosa. In contrast, ADSC-EV intervention significantly attenuated neutrophil infiltration, which might correlate with mucosal architecture and epithelial damage restoration [Figure 5B]. To clarify the characteristics of intrahepatic inflammation, we analyzed the subset distribution of infiltrated monocyte-derived macrophages (MoMFs) in liver tissue. Two functionally distinct macrophage subsets were focused on: CD45^+^-Ly6G^-^-CD11c^-^-CD3^-^-CD11b^+^-F4/80^low^-Ly6c^high^ (representing infiltrated pro-inflammatory/fibrotic MoMFs) and CD45^+^-Ly6G^-^-CD11c^-^-CD3^-^-CD11b^+^-F4/80^low^-Ly6c^low^ (representing infiltrated anti-inflammatory/restorative MoMFs). The results showed that the proportion of the Ly6c^high^ subset among MoMFs was significantly higher in the LF group than in the Sham group, while ADSC-EV intervention notably reduced this proportion. Not surprisingly, the proportion of Ly6c^low^ subset exhibited an opposite trend [Figure 5C]. We also quantitatively analyzed the Ly6c^high^/Ly6c^low^ ratio to evaluate the hepatic inflammatory state. The findings suggested that ADSC-EV intervention could significantly reduce the Ly6c^high^/Ly6c^low^ ratio compared with the LF group [Figure 5D], indicating that the inflammatory state shifted from pro-inflammatory/pro-fibrotic to an anti-inflammatory/restorative state. Together, these results demonstrate that ADSC-EVs induce butyric acid level elevation following the gut microbiota reshape and alleviate systemic inflammation in LF mice.

## DISCUSSION

LF primarily arises from chronic hepatic injury triggered by persistent insults, including viral hepatitis [e.g., hepatitis B virus (HBV), hepatitis C virus (HCV)], excessive alcohol consumption, metabolic dysfunction-associated steatohepatitis (MASH), cholestatic diseases, and autoimmune disorders, which initiate a sustained wound-healing response^[29]^. These pathological characteristics of LF are the excessive accumulation of ECM, predominantly fibrillar collagens, driven by the activation and trans-differentiation of HSCs into myofibroblasts. This process leads to progressive architectural distortion, bridging fibrosis, and ultimately nodule formation in cirrhosis^[30]^. Globally, hundreds of millions of individuals are affected by LF to varying degrees. Significant fibrosis (≥ F2) is estimated to affect over 500 million people, while advanced fibrosis or cirrhosis affects more than 100 million^[31]^. The burden is still rising rapidly, due to the global epidemics of obesity, diabetes, and MASH.

At present, clinical management of LF remains reliant on etiology control (e.g., antiviral therapies, metabolic interventions). There is a lack of globally approved direct anti-fibrotic drugs. Pan-fibrotic agents such as pirfenidone are plagued by significant off-target toxicity and low liver-specific efficacy^[32]^. While the approval of resmetirom for MASH-associated fibrosis represents a pivotal advance, real-world data imply that liver biopsy is still required to screen eligible patients and assess its therapeutic potential and prognostic value^[33,34]^. Furthermore, glucagon-like peptide-1 (GLP-1)-based therapies (e.g., Semaglutide, Survodutide) are only beneficial for metabolism-associated fibrosis^[35]^. Therefore, effective and comprehensive anti-fibrotic strategies that address the heterogeneous etiologies are still lacking.

In chronic liver injury, intestinal barrier dysfunction is a common sequela. Disruption of tight junctions and destruction of mucus layer integrity are caused by factors such as bile acid (BA) deficiency, portal hypertension-induced edema, and systemic inflammation, increasing gut permeability, commonly known as intestinal leakage^[36]^. Increased intestinal permeability facilitates the translocation of PAMPs and damage-associated molecular patterns (DAMPs) into the portal circulation. These molecules can activate hepatic inflammatory pathways such as toll-like receptor 4 (TLR4)/nucleotide oligomerization domain-like receptor family, pyrin domain containing 3 (NLRP3), thereby inducing pro-inflammatory and pro-fibrotic cytokine release [e.g., tumor necrosis factor (TNF)-α, interleukin (IL)-1β, TGF-β] that facilitate HSC activation and ECM deposition, ultimately establishing a self-amplifying “gut-liver axis” of injury^[37]^.

Because the intestine and liver are closely connected through the “gut-liver axis”, the substances absorbed by the intestine (including the microbiota and its metabolites) are directly transported to the liver via the portal vein. Consequently, the imbalance of gut microbiota and the dysregulated changes in their metabolites have also emerged as core mechanisms driving the progression of LF. Firstly, during LF progression, the gut microbiota exhibits an overall decrease in species richness and evenness. Secondly, it undergoes compositional alterations. The microbial dysbiosis manifests as a depletion of beneficial bacteria, including *Lactobacillus*, *Bifidobacterium*, *etc.*, while an increase in the abundance of conditionally pathogenic bacteria with pro-inflammatory potential, such as *Enterobacteriaceae*, *Streptococcaceae*, *etc.* Several studies have reported that the decrease of *Firmicutes/Bacteroidetes* (F/B) and *Firmicutes/Proteobacteria* (F/P) ratios is associated with poor prognosis in patients with LF and cirrhosis^[38,39]^. Thirdly, microbial metabolic function is disrupted, primarily due to a decline in SCFAs produced by microbial communities. The levels of crucial SCFAs such as acetate, propionate and butyrate with anti-inflammatory and barrier protective functions were decreased in the intestine. Conversely, excessive proliferation of conditional pathogens such as *Enterobacteriaceae*, *Enterococcus*, and *Streptococcus* may exacerbate liver inflammation by directly or indirectly increasing lipopolysaccharide (LPS), cytolysin and reactive oxygen species (ROS) levels. Therefore, effective therapeutic strategies for LF not only exert direct hepato-protective and anti-fibrotic effects but also restore intestinal barrier integrity and maintain microbial homeostasis to disrupt the pathological gut-liver axis cycle. There is an urgent need for a novel strategy to target this dual pathway.

EVs, as a promising cell-free therapy, critically mediate intercellular communication by transferring bioactive molecules such as miRNAs, proteins, and lipids. During liver injury, these molecules can modulate the crosstalk among hepatocytes, immune cells, HSCs and endothelial cells, thereby regulating key pathological processes including inflammation, apoptosis, oxidative stress, and fibrogenesis. In our previous study, we found that ADSCs-derived EVs significantly improved the survival rate from 25% to over 70% in the acute liver failure rat model by releasing the lncH19 and inhibiting hepatocytes apoptosis^[18]^. A recent study also demonstrated that ADSC-EVs with augmenter of liver regeneration (ALR) over-expressed could protect the injured liver from apoptosis, regeneration suppression, cellular mitochondrial structure and function dysregulation^[40]^. Another research reported that ADSC-EVs could reduce the pyroptosis in injured liver and promote liver regeneration-associated factors expression^[41]^. These studies supported that ADSC-EVs represent a promising cell-free therapeutic strategy for combating liver injury and its progression to fibrosis or cirrhosis.

In this study, we isolated and purified ADSC-EVs that exhibited classical morphology, size and EV markers. Subsequent experiments demonstrated that ADSC-EV treatment exerted significant therapeutic effects in the DEN/CCl_4_-induced LF mouse model, including improving liver function, reducing excessive ECM deposition and suppressing HSC activation. To further elucidate the protective potential of ADSC-EVs against gut-liver axis dysregulation, we then evaluated intestinal function and gut microbiota in LF mice. Our findings showed that ADSC-EV treatment significantly restored intestinal barrier integrity by reducing intestinal permeability and reinforcing the mucus barrier. More importantly, ADSC-EV treatment modulated the gut microbiota dysbiosis, characterized by an increase in beneficial genera, including *Akkermansia* and *Lachnospiraceae NK4A136-group*, with a particularly significant elevation in the abundance of *A. muciniphila*. We believed that the intestinal barrier repair might effectively limit bacterial translocation, thereby disrupting the pathogenic gut-liver crosstalk. Additionally, ADSC-EV-induced alterations in the microbiota may further contribute to a better intestinal microenvironment to support gut barrier health, forming a virtuous cycle. Based on this hypothesis, we detected increased butyric acid levels in cecal content and a significant reduction in systemic inflammation. Based on current data, we tended to believe that the improvement of microbial homeostasis was the sum of direct and indirect effects of ADSC-EVs. Improved liver function and an intact intestinal barrier could reduce bacterial translocation by reducing systemic inflammation, altering BA profiles, and creating a favorable intestinal microenvironment for beneficial microorganisms, then indirectly promoting the recovery of microbial homeostasis. A quantitative biodistribution and pharmacokinetics study of exosomes labeled with ^89^Zr radioisotope in mice and rats reported that ^89^Zr-labeled exosomes (^89^Zr-Exo) were rapidly internalized by cells and tissues within 15 min and mainly distributed in the liver and spleen. At approximately 6 h of administration, ^89^Zr-Exo appeared in the intestine and stomach with a peak intensity^[42]^.

Recent studies suggested that *A. muciniphila* played a vital role in strengthening intestinal barrier integrity, mitigating inflammation through multiple potential mechanisms such as inhibiting the adenosine 5’-monophosphate (AMP)-activated protein kinase (AMPK) pathway, nucleotide-binding domain (NOD)-like receptor family and NLRP3 activation^[25]^. Emerging evidence has also demonstrated that a reduced abundance of *A. muciniphila* is significantly correlated with the progression of LF. *A. muciniphila* acted as a beneficial commensal bacterium in liver injury disease^[43]^. Crucially, several studies have shown that *A. muciniphila* can promote the growth of butyrate-producing bacteria by supplying essential metabolites that facilitate their growth and metabolic activity within the gut environment^[44,45]^. Furthermore, butyric acid serves as the primary energy source for colonic epithelial cells, directly enhancing tight junction assembly, stimulating mucus production by goblet cells, and exerting anti-inflammatory effects. Thus, the increased butyric acid levels synergize with the enriched *A. muciniphila* to facilitate gut barrier restoration.

Therefore, ADSC-EVs orchestrated a multi-faceted restoration of gut homeostasis, modulating the gut microbiota by enriching barrier-promoting commensal bacteria *A. muciniphila*, then increasing beneficial metabolites butyric acid, and consequently enhancing intestinal barrier integrity. This collective action disrupts the vicious cycle of the liver-gut axis, ultimately contributing significantly to the alleviation of LF. For other intestinal diseases such as inflammatory bowel disease (IBD), Crohn’s disease (CD), and ulcerative colitis (UC), natural and modified therapeutic EVs can also be taken up by intestinal cells and bacteria to relieve gastrointestinal injury through immunomodulation and restoration of intestinal homeostasis, which may provide a broad clinical application prospect^[46]^. Notably, this study has several limitations that should be acknowledged. Although we demonstrated the beneficial effects of ADSC-EVs on liver function, gut barrier integrity, and the microbial community, our findings are still limited to correlative analyses of the “ADSC-EVs–gut–liver” axis. In this research, our data suggested an upregulatory effect of ADSC-EVs on the probiotic *A. muciniphila* in gut microbiota; however, the direct influence of ADSC-EVs on *A. muciniphila* colonization and its metabolic mechanism remains to be fully elucidated by a germ-free mice model and metabolomics. In particular, in future research, we would use gas chromatography-mass spectrometry (GC-MS) or liquid chromatography-mass spectrometry (LC-MS) for absolute quantification of SCFAs (including butyric acid, acetic acid, propionic acid, *etc.*) in intestinal contents. This will support the precise study of causal relationships and molecular mechanisms. Moreover, the therapeutic ADSC-EVs administered in this study were native and non-targeted EVs, resulting in a liver-dominated biodistribution profile with limited direct delivery to intestinal tissue. Therefore, it is crucial to develop gut-specific, engineered EVs to maximize local effects and functional potency in future studies.

## DECLARATIONS

### Availability of data and materials

Additional data related to this article may be obtained from the corresponding author upon reasonable request.

### Copyright

© The Author(s) 2025.
